# DNA Repair Gene XRCC1 Polymorphisms and Head and Neck Cancer Risk: An Updated Meta-Analysis Including 16344 Subjects

**DOI:** 10.1371/journal.pone.0074059

**Published:** 2013-09-23

**Authors:** Yin Lou, Wen-jia Peng, Dong-sheng Cao, Juan Xie, Hong-hong Li, Zheng-xuan Jiang

**Affiliations:** 1 Department of Plastic Surgery, The second Hospital of Anhui Medical University, Hefei, China; 2 Department of Epidemiology and Biostatistics, School of Public Health, Anhui Medical University, Hefei, China; 3 Department of Ophthalmology, The second Hospital of Anhui Medical University, Hefei, China; University of Hawaii Cancer Center, United States of America

## Abstract

**Background:**

DNA repair gene X-ray repair cross complementing group 1 (XRCC1) plays an important role in the maintenance of the genomic integrity and protection of cells from DNA damage. Sequence variation in XRCC1 gene may alter head and neck cancer (HNC) susceptibility. However, these results are inconclusive. To derive a more precise estimation of the relationship between XRCC1 polymorphism and HNC risk, we undertook a meta-analysis involving 16,344 subjects.

**Methods:**

A search of the literature by PubMed, Embase, Web of Science and China National Knowledge Infrastructure was performed to identify studies based on the predetermined inclusion criteria. The odds ratio (OR) with 95% confidence interval (CI) was combined using a random-effects model or a fixed-effects model.

**Results:**

Twenty-nine studies consisting of 6,719 cases and 9,627 controls were identified and analyzed. Overall, no evidence of significant association was observed between XRCC1 Arg194Trp, XRCC1 Arg280His, XRCC1 Arg399Gln genotypes and the risk of HNC in any genetic models. Subgroup analyses according to ethnicity, tumor site, publication year, genotyping method also detected no significant association in any subgroup, except that oral cancer was associated with Arg194Trp variant in recessive model. Furthermore, no significant effect of these polymorphisms interacted with smoking on HNC risk was detected but Arg194Trp homozygous variant.

**Conclusion:**

In conclusion, this meta-analysis suggests that the XRCC1 Arg194Trp, Arg280His and Arg399Gln polymorphism may not involve in HNC susceptibility. Further studies about gene-gene and gene-environment interactions in different populations are required.

## Introduction

Head and neck cancer (HNC) is now the fifth most common type of cancer in the world [1], with approximately 434,000 new patients diagnosed annually worldwide [2]. Most of the cases involving new patients occur in economically developing countries, such as India, Brazil, and Thailand [3,4]. HNC is generally divided into three groups: oral cavity, pharynx, and larynx. Evolvement of HNC is a multifactorial process associated with various risk factors. Accumulative evidence indicates that tobacco smoking, drinking alcohol, and chewing betel quid are three major risk factors for HNC [5,6]. These environmental carcinogens may induce a defective DNA damage response, which may lead to apoptosis or may result in genomic instability and un-regulated (proliferative) cell growth [7–9].

The DNA repair system aims to maintain genomic integrity, and constantly challenge the environmental insults and replication errors. Therefore, the alteration of DNA repair genes could increase the risk of carcinoma in the head and neck [10]. Three important DNA repair pathways, including nucleotide excision repair (NER), base excision repair (BER), and double strand break (DSB), are involved in this process. The x-ray repair cross-complementing group 1 (XRCC1) involved in the BER pathway is thought to play a key role in protecting the genome from a variety of risk factors. Three common single nucleotide polymorphisms in the XRCC1 gene, including Arg194Trp (C to T substitution at exon 6 resulting in an Arg to Trp amino acid change), Arg280His (G to A substitution at exon 9 resulting in an Arg to His amino acid change), and Arg399Gln (G to A substitution at exon 10 resulting in an Arg to Gln amino acid change) are most commonly tested in many studies that have examined different populations.

Multiple studies have evaluated the association of HNC risk with polymorphism in the DNA repair genes XRCC1 Arg194Trp, XRCC1 Arg280His, and XRCC1 Arg399Gln. However, these results are inconsistent. While no association between XRCC1 polymorphisms and HNC risk was demonstrated in some studies [11,12], but Ramachandran et al. [13] and Olshan et al. [14] found a relationship between XRCC1 Arg194Trp and Arg399Gln polymorphisms and the risk of HNC. Olshan et al. [14] performed a stratified analysis to estimate the interaction between XRCC1 polymorphisms and smoking, suggesting that the Arg194Trp and Arg399Gln variants of XRCC1 were associated with the risk of HNC in those cases, but no association was found in Kumar’s research [15]. Although Flores-Obando et al. [16] performed a meta-analysis in 2010 on the relationship between XRCC1 polymorphisms and the risk of HNC, subgroup analyses of smoking and genotyping method were not performed. Considering these conflicting results, we conducted an updated meta-analysis to deduce a reasonable conclusion about the relationship between XRCC1 polymorphisms and HNC risk. Subgroup analyses concerning ethnicity, smoking, site of HNC, publication year, and genotyping method were performed. Therefore, the current meta-analysis has a greater ability power to derive a more accurate conclusions than previous meta-analyses.

## Materials and Methods

### Search strategy

A systematic and electronic search of the PubMed, EMBASE, Web of Science, and China National Knowledge Infrastructure (CNKI) databases was performed to identify studies using combinations of the following search terms: “head and neck”, “oral”, “pharynx”, “larynx”, “nasopharynx”, “cancer”, “tumor”, “carcinoma”, “x-ray repair cross complementing group 1”, “XRCC1”, “Arg194Trp”, “Arg280His”, “Arg399Gln”, “polymorphism”, and “variation”. All of the studies were published from their earliest entry points to March 2013.

### Selection

All of the studies met the following inclusion criteria: (1) published in English; (2) examined case-control studies estimating the relationship between XRCC1 polymorphism and the risk of HNC; (3) described genotype frequencies; (4) genotype distribution in controls must be in Hardy-Weinberg equilibrium (HWE); and (5) when duplicated studies were published by the same author obtained from the same patient sample, only the most complete publication study was included in this meta-analysis. Unpublished reports and abstracts were not considered.

### Data extraction

The data were collected according to a standard protocol. The following information was extracted from each study: name of the first author, year of publication, country, genotyping methods, ethnicity and source of the cases and controls, characteristics of the sample population, and the genotype numbers from the cases and the controls.

### Statistical analysis

We first tested for deviations from the Hardy-Weinberg equilibrium (HWE) in the control groups using the goodness-of-fit test (Chi-square test or Fisher exact test). The odds ratio (OR) with a corresponding 95% confidence interval (CI) was used to examine the association between XRCC1 polymorphism and HNC risk. The current meta-analysis used the following statistical models, the allelic genetic model, the codominant genetic model (homozygote comparison), and the recessive genetic model. Heterogeneity among the studies was assessed using the chi-square-based Q statistic (P<0.1 for the Q test indicates significant heterogeneity) [17]. We also quantified the effect of heterogeneity using the I^2^ statistic [18]. Either the random-effects model (DerSimonian-Laird method [19]) or the fixed-effects model (Mantel-Haenszel method [20]) was used to calculate pooled effect estimates in the presence or absence of heterogeneity, respectively. Finally, potential publication bias was evaluated through Begg’s test and Egger’s test by visual analysis of the funnel plot [21,22]. P< 0.05 was considered statistically significant publication bias. Genotype frequencies in the control populations according to race were calculated and tests on the equality of proportions was performed for the Asian and Caucasian control populations in order to compare differences in genotype frequencies between the two groups. All statistical analyses were performed with the STATA version 10.0 software (Stata Corporation, College Station, TX).

## Results

### Studies characteristics

**Figure 1:**
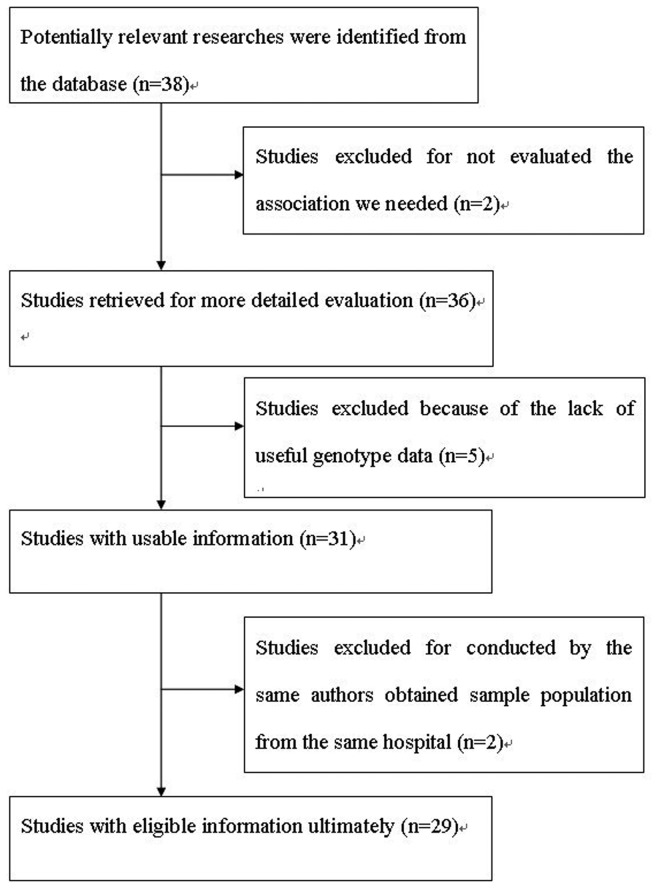
Flow diagram of articles selection process.

As shown in Figure 1, the computerized search using the search strategy mentioned above delivered 38 publications. Of these, two papers were excluded due to the fact that they did not evaluate the association between HNC risk and XRCC1 polymorphisms [23,24]. Subsequently, five studies were excluded because of the lack of useful genotype data [25–29]. In the remaining 31 studies, two papers were excluded because of overlapping data [30,31]. Ultimately, 29 studies were identified as eligible and they were analyzed [11–15,32–55].

In total, 29 reports, consisting of 6,719 cases and 9,627 controls, matching the inclusion criteria were included in the present meta-analysis. The characteristics are summarized in Table 1. Of those 29 reports, 15 studies were performed on Caucasians, 10 studies were performed on Asians, and four studies were performed on a mixed population. In the 29 studies, 23 focused on the relationship between XRCC1 Arg194Trp polymorphism and HNC risk, 11 focused on Arg280His polymorphism, and 28 investigated the association between Arg399Gln polymorphism and HNC risk. In 19 studies, the controls were from a healthy population and in eight studies the controls were from a hospital population.

**Table 1:** Main characteristics of studies included in the meta-analysis.

First author (year)	Country	Ethnicity	Control source	Tumor Sites	Genotyping Methods	Sample size (case/control)	Research of environmental factors
Sturgis et al.(1999)	USA	Caucasian	Hospital	Oral cavity, larynx, oro/hypo-pharynx	PCR-RFLP	203/424	NR
Olshan et al.(2002)	USA	Caucasian	Hospital	Oral cavity, larynx, pharynx	PCR-RFLP	98/161	Smoking
Varzim et al.(2003)	Portugal	Caucasian	Healthy	Larynx	PCR-RFLP	88/178	NR
Cho et al.(2003)	Taiwan	Asian	Healthy	Nasopharynx	PCR-RFLP	334/282	NR
Tae et al.(2004)	Korea	Asian	Hospital	Oral cavity, larynx, oro/hypo-pharynx	Sequence	129/157	NR
Demokan et al.(2005)	Turkey	Other	Healthy	NR	PCR-RFLP	95/98	Smoking, alcohol
Matullo et al.(2005)	Europe	Caucasian	Healthy	Oral cavity, larynx, pharynx	Taqman	82/1094	Smoking
Rydzanicz et al.(2005)	Poland	Caucasian	Healthy	Oral cavity, tongue, larynx and pharynx	PCR-RFLP	182/143	Smoking
Gajecka et al.(2005)	Poland	Caucasian	Healthy	Larynx	PCR-RFLP	293/319	NR
Kietthubthew et al. (2006)	Thailand	Asian	Healthy	Oral cavity	PCR-RFLP	106/164	Smoking
Ramachandran et al. (2006)	India	Asian	Hospital	Oral cavity	PCR-RFLP	110/110	Smoking, alcohol, betel quid chewing
Cao et al.(2006)	China	Asian	Healthy	Nasopharynx	PCR-RFLP	425/501	Smoking
Li et al.(2007)	USA	Caucasian	Healthy	Oral cavity, larynx, pharynx	PCR-RFLP	830/854	Smoking, alcohol
Majumder et al.(2007)	India	Asian	Hospital	Oral cavity	PCR-RFLP	309/385	NR
Yang et al.(2007)	China	Asian	Healthy	Nasopharynx	PCR-RFLP	153/168	NR
Ho et al.(2007)	USA	Caucasian	Hospital	Oral cavity	PCR-RFLP	138/503	NR
Harth et al.(2008)	Germany	Caucasian	Hospital	Oral cavity, larynx, pharynx	PCR-RFLP	310/300	Smoking
Yen et al.(2008)	Taiwan	Asian	Hospital	Oral cavity	PCR-RFLP	103/98	NR
Csejtei et al.(2009)	Hungary	Caucasian	Healthy	Oral cavity, larynx, pharynx	PCR-RFLP	108/102	Smoking
Kowalski et al.(2009)	Poland	Caucasian	Healthy	Oral cavity, larynx, pharynx	PCR-RFLP	92/124	Smoking
Applebaum et al. (2009)	USA	Caucasian	Healthy	Oral cavity, larynx, oro/hypo-pharynx	PCR-RFLP	483/547	Smoking
Jelonek et al.(2010)	Poland	Caucasian	Healthy	NR	PCR-RFLP	104/252	NR
Gugatschka et al.(2011)	Austria	Caucasian	Healthy	NR	Taqman	168/463	NR
Laantri et al.(2011)	Morocco	African	NR	Nasopharynx	Taqman	512/477	NR
Kumar et al.(2012)	India	Asian	Healthy	Oral cavity, tongue, larynx and pharynx	PCR-RFLP	278/278	Smoking, alcohol, tobacco chewing
Yuan et al.(2012)	China	Asian	Healthy	Oral cavity, larynx, oropharynx	Taqman	390/886	NR
Al-Hadyan et al. (2012)	Saudi Arabia	Other	Healthy	Nasopharynx	Sequence	156/251	NR
Dos Reis et al.(2012)	Brazil	Other	Healthy	Oral cavity	PCR-RFLP	150/150	NR
Kostrzewska-Poczekaj et al.(2012)	Poland	Caucasian	NR	Oral cavity, larynx	PCR-RFLP	290/158	NR

Abbreviations:NR= not reported; PCR-RFLP= PCR-based restriction fragment length polymorphism

The distribution of XRCC1 Arg194Trp, XRCC1 Arg280His, and XRCC1 Arg399Gln polymorphism genotype frequencies between the HNC cases and the controls in the 29 studies are shown in Table 2. Noticeably, genotype distribution in the controls of Arg194Trp polymorphism in the study by Demokan et al. [36] and Arg399Gln polymorphism in the study by Dos Reis et al. [46] deviate from HWE, which are excluded in the subgroup analyses.

**Table 2:** Distribution of XRCC1 genotypes among head and neck cancer cases and controls included in the meta-analysis.

Gene Polymorphism	First author (year)	Cases (n)			Controls (n)			P-value of HWE in controls
XRCC1-Arg194Trp		Arg/Arg	Arg/Trp	Trp/Trp	Arg/Arg	Arg/Trp	Trp/Trp	
	Sturgis et al. (1999)	180	22	1	363	61	0	0.279
	Olshan et al. (2002)	82	16	0	135	26	0	0.537
	Varzim et al. (2003)	80	8	0	160	18	0	0.777
	Tae et al. (2004)	59	52	9	101	39	5	0.879
	Matullo et al. (2005)	78	4	0	951	141	2	0.391
	Rydzanicz et al. (2005)	165	16	1	129	14	0	0.827
	Gajecka et al. (2005)	262	27	1	291	33	1	0.998
	Kietthubthew et al. (2006)	40	50	16	77	67	20	0.664
	Ramachandran et al. (2006)	66	37	7	90	19	1	0.999
	Cao et al. (2006)	232	166	19	235	217	43	0.776
	Majumder et al. (2007)	248	58	3	317	62	8	0.074
	Yang et al. (2007)	62	79	12	99	65	4	0.204
	Ho et al. (2007)	108	29	0	433	69	1	0.592
	Harth et al. (2008)	217	40	1	259	39	2	0.924
	Yen et al. (2008)	48	40	15	54	35	9	0.643
	Csejtei et al. (2009)	96	11	1	85	15	2	0.425
	Kowalski et al. (2009)	71	21	0	102	22	0	0.556
	Applebaum et al. (2009)	427	55	2	485	61	3	0.776
	Gugatschka et al. (2011)	148	20	0	397	63	3	0.959
	Laantri et al. (2011)	492	55	4	470	41	1	0.994
	Kumar et al. (2012)	144	111	23	121	131	26	0.535
	Dos Reis et al. (2012)	127	23	0	123	24	3	0.396
XRCC1-Arg280His		Arg/Arg	Arg/His	His/His	Arg/Arg	Arg/His	His/His	
	Cho et al. (2003)	275	55	2	215	66	2	0.442
	Tae et al. (2004)	113	21	1	139	29	0	0.473
	Ramachandran et al. (2006)	77	31	2	83	26	1	0.798
	Majumder et al. (2007)	225	79	3	297	87	3	0.461
	Yang et al. (2007)	125	27	1	131	35	2	0.981
	Ho et al. (2007)	125	13	0	453	50	0	0.503
	Harth et al. (2008)	283	28	1	270	30	0	0.660
	Applebaum et al. (2009)	437	46	1	492	52	4	0.150
	Gugatschka et al. (2011)	159	9	0	430	32	1	0.885
	Laantri et al. (2011)	431	114	10	405	92	9	0.382
	Kumar et al. (2012)	129	123	26	142	116	20	0.855
XRCC1- Arg399Gln		Arg/Arg	Arg/Gln	Gln/Gln	Arg/Arg	Arg/Gln	Gln/Gln	
	Sturgis et al. (1999)	94	77	32	181	197	46	0.782
	Olshan et al. (2002)	45	50	3	62	82	17	0.412
	Varzim et al. (2003)	37	40	11	80	80	18	0.954
	Cho et al. (2003)	174	128	32	152	109	21	0.972
	Tae et al. (2004)	69	51	9	86	64	7	0.517
	Demokan et al. (2005)	42	41	12	39	46	13	0.995
	Matullo et al. (2005)	34	38	10	484	482	128	0.892
	Rydzanicz et al. (2005)	63	98	21	59	63	21	0.825
	Gajecka et al. (2005)	106	153	34	124	145	50	0.783
	Kietthubthew et al. (2006)	55	45	6	67	74	23	0.940
	Ramachandran et al. (2006)	46	48	16	73	33	4	0.996
	Cao et al. (2006)	241	152	32	270	201	30	0.651
	Li et al. (2007)	335	374	121	360	385	109	0.929
	Majumder et al. (2007)	134	143	32	170	179	36	0.523
	Yang et al. (2007)	93	54	6	95	67	6	0.370
	Ho et al. (2007)	61	62	15	220	216	67	0.486
	Harth et al. (2008)	114	166	30	143	121	36	0.423
	Csejtei et al. (2009)	50	47	11	53	41	8	0.999
	Kowalski et al. (2009)	37	44	11	49	53	22	0.521
	Applebaum et al. (2009)	192	229	62	232	246	69	0.956
	Jelonek et al.(2010)	47	50	7	103	124	25	0.374
	Gugatschka et al. (2011)	70	74	24	204	198	61	0.503
	Laantri et al. (2011)	274	193	45	279	163	35	0.268
	Kumar et al. (2012)	128	124	26	98	144	36	0.323
	Yuan et al. (2012)	221	146	23	481	339	66	0.842
	Al-Hadyan et al. (2012)	96	50	10	135	99	17	0.980
	Kostrzewska-Poczekaj et al. (2012)	110	154	26	50	81	27	0.837
Arg194Trp influenced by smoking		Arg/Arg	Arg/Trp	Trp/Trp	Arg/Arg	Arg/Trp	Trp/Trp	
	Olshan et al. (2002)	74	16	0	81	16	0	0.675
	Rydzanicz et al. (2005)	165	16	1	129	14	0	0.827
	Cao et al. (2006)	154	108	9	78	62	14	0.947
	Csejtei et al. (2009)	96	11	1	85	15	2	0.425
	Kowalski et al. (2009)	49	17	0	44	8	0	0.835
Arg399Gln influenced by smoking		Arg/Arg	Arg/Gln	Gln/Gln	Arg/Arg	Arg/Gln	Gln/Gln	
	Rydzanicz et al. (2005)	63	98	21	59	63	21	0.825
	Cao et al. (2006)	156	102	21	85	60	12	0.953
	Csejtei et al. (2009)	50	47	11	53	41	8	0.999
	Kowalski et al. (2009)	19	36	11	36	16	0	0.423

Abbreviations: HWE= Hardy–Weinberg equilibrium.

### Meta-analysis results

The overall results of the meta-analysis for XRCC1 polymorphism and the risk of HNC are shown in Table 3.

**Table 3:** Results of meta-analysis for XRCC1 polymorphism and the risk of HNC.

Comparison	Number of studies	Sample size (case/control)	Test of association				Test of heterogeneity			Publication bias	
			OR	95%CI	P value	Model	Q	P value	I^2^	P value (Begg’s)	P value (Egger’s)
**Arg194Trp**											
*Arg194 allele vs. Trp194 allele*											
Total	22	4,478/6,873	0.91	0.77-1.08	0.279	R	66.78	0.000	68.6%	0.367	0.449
Caucasian	12	2,190/4,366	1.04	0.89-1.21	0.652	F	12.28	0.304	10.4%	0.086	0.108
Asian	8	1,596/1,845	0.76	0.55-1.05	0.095	R	50.48	0.000	86.1%	0.019	0.001
OC	6	915/1,412	0.74	0.55-1.01	0.054	R	13.99	0.016	64.3%	0.452	0.573
Smoking	5	717/548	1.19	0.94-1.52	0.155	F	4.83	0.305	17.2%	0.221	0.219
Publication year	4	1147/1403	1.11	0.93-1.34	0.251	F	5.95	0.114	49.6%	1.000	0.890
PCR-RFLP	18	3566/4659	0.92	0.77-1.09	0.332	R	49.24	0.000	65.5%	0.820	0.176
Taqman	3	801/2069	1.21	0.63-2.35	0.768	R	7.65	0.022	73.9%	0.296	0.221
*Arg/Arg vs. Trp/Trp*											
Total	22	4,478/6,873	0.80	0.50-1.28	0.349	R	33.77	0.013	46.7%	0.944	0.245
Caucasian	12	2,190/4,366	1.04	0.45-2.40	0.920	F	2.95	0.937	0.0%	0.118	0.125
Asian	8	1,596/1,845	0.70	0.37-1.35	0.294	R	27.71	0.000	74.7%	0.035	0.046
OC	6	915/1,412	0.71	0.44-1.13	0.145	F	8.40	0.136	40.4%	1.000	0.715
Smoking	5	717/548	2.53	1.16-5.53	0.020	F	1.38	0.502	0.0%	0.296	0.346
Publication year	4	1147/1403	1.33	0.77-2.28	0.308	F	3.55	0.314	15.5%	0.308	0.908
PCR-RFLP	18	3566/4659	0.90	0.54-1.50	0.684	R	27.70	0.016	49.5%	0.621	0.334
Taqman	3	801/2069	0.59	0.16-2.22	0.439	F	1.55	0.461	0.0%	1.000	0.525
*Arg/Arg vs.Arg/Trp+ Trp/Trp*											
Total	22	4,478/6,873	0.90	0.75-1.08	0.225	R	61.79	0.000	66.0%	0.693	0.450
Caucasian	12	2,190/4,366	1.03	0.88-1.21	0.711	F	13.22	0.279	16.8%	0.086	0.112
Asian	8	1,596/1,845	0.72	0.50-1.06	0.094	R	45.55	0.000	84.6%	0.019	0.003
OC	6	915/1,412	0.70	0.52-0.95	0.022	R	10.66	0.059	53.1%	1.000	0.483
Smoking	6	842/705	1.57	0.68-3.64	0.289	R	44.81	0.000	88.8%	0.452	0.948
Publication year	4	1147/1403	1.13	0.91-1.40	0.283	F	5.53	0.137	45.7%	0.734	0.852
PCR-RFLP	18	3566/4659	0.90	0.75-1.10	0.305	R	44.93	0.000	62.2%	0.940	0.156
Taqman	3	801/2069	1.22	0.63-2.33	0.704	R	6.88	0.032	70.9%	0.296	0.169
**Arg280His**											
*Arg280 allele vs. His280 allele*											
Total	11	2,972/3,714	0.98	0.87-1.10	0.757	F	9.87	0.452	0.0%	0.276	0.153
Caucasian	4	1,102/1,804	1.12	0.86-1.45	0.411	F	0.42	0.937	0.0%	0.734	0.508
Asian	6	1,315/1,394	0.97	0.83-1.13	0.696	F	8.00	0.156	37.5%	0.452	0.550
OC	3	555/1,000	0.86	0.67-1.10	0.241	F	0.59	0.746	0.0%	1.000	0.749
Publication year	3	1001/1247	0.89	0.75-1.07	0.220	F	1.48	0.477	0.0%	0.296	0.097
PCR-RFLP	8	2114/2577	0.99	0.87-1.14	0.922	F	8.48	0.292	17.4%	0.711	0.413
Taqman	2	723/969	0.94	0.73-1.20	0.617	F	1.21	0.272	17.2%	1.000	
*Arg/Arg vs.His/His*											
Total	11	2,972/3,714	0.84	0.55-1.29	0.427	F	3.73	0.928	0.0%	0.721	0.638
Caucasian	4	1,102/1,804	1.56	0.38-6.41	0.536	F	1.42	0.492	0.0%	1.000	0.276
Asian	6	1,315/1,394	0.74	0.44-1.24	0.250	F	1.44	0.920	0.0%	0.707	0.826
OC	3	555/1,000	0.65	0.17-2.44	0.521	F	0.11	0.741	0.0%	1.000	
Publication year	3	1001/1247	0.78	0.47-1.30	0.342	F	0.36	0.836	0.0%	1.000	0.559
PCR-RFLP	8	2114/2577	0.83	0.50-1.37	0.463	F	3.13	0.792	0.0%	1.000	0.404
Taqman	2	723/969	0.97	0.40-2.32	0.943	F	0.01	0.930	0.0%	1.000	
*Arg/Arg vs. Arg/His + His/His*											
Total	11	2,972/3,714	0.99	0.87-1.13	0.872	F	9.95	0.445	0.0%	0.276	0.205
Caucasian	4	1,102/1,804	1.10	0.84-1.44	0.483	F	0.34	0.952	0.0%	0.308	0.258
Asian	6	1,315/1,394	0.99	0.83-1.18	0.913	F	8.26	0.142	39.5%	0.452	0.680
OC	3	555/1,000	0.85	0.65-1.12	0.247	F	0.61	0.736	0.0%	1.000	0.746
Publication year	3	1001/1247	0.88	0.72-1.09	0.252	F	1.38	0.502	0.0%	1.000	0.200
PCR-RFLP	8	2114/2577	1.01	0.86-1.17	0.938	F	8.41	0.298	16.8%	0.536	0.596
Taqman	2	723/969	0.92	0.70-1.21	0.565	F	1.16	0.282	13.7%	1.000	
**Arg399Gln**											
*Arg399 allele vs. Gln399 allele*											
Total	27	6,466/9,379	1.01	0.94-1.09	0.850	R	50.97	0.002	49.0%	0.532	0.529
Caucasian	14	2,639/4,768	1.00	0.93-1.08	0.965	F	14.09	0.368	7.7%	0.511	0.324
Asian	9	2,234/2,931	1.01	0.84-1.21	0.931	R	30.42	0.000	73.7%	0.466	0.425
OC	4	663/1,162	0.91	0.59-1.40	0.674	R	22.41	0.000	86.6%	1.000	0.685
Smoking	4	635/454	0.70	0.43-1.15	0.158	R	18.19	0.000	83.5%	0.089	0.042
Publication year	7	1898/2765	1.12	0.96-1.29	0.149	R	14.08	0.029	57.4%	0.764	0.276
PCR-RFLP	21	5029/6051	1.02	0.93-1.11	0.737	R	44.77	0.001	55.3%	0.566	0.558
Taqman	4	1152/2920	0.96	0.85-1.08	0.478	F	3.73	0.292	19.6%	1.000	0.762
Sequence	2	285/408	1.08	0.85-1.39	0.519	F	1.56	0.212	35.8%	1.000	
*Arg/Arg vs.Gln/Gln*											
Total	27	6,466/9,379	1.03	0.88-1.20	0.714	R	43.00	0.019	39.5%	0.260	0.330
Caucasian	14	2,639/4,768	1.08	0.92-1.28	0.348	F	16.57	0.219	21.6%	0.324	0.157
Asian	9	2,234/2,931	0.97	0.65-1.44	0.874	R	22.86	0.004	65.0%	0.466	0.478
OC	4	663/1,162	0.91	0.38-2.20	0.838	R	15.87	0.001	81.1%	0.806	0.692
Smoking	4	635/454	0.73	0.33-1.63	0.445	R	7.49	0.058	60.0%	0.089	0.002
Publication year	7	1898/2765	1.28	0.93-1.75	0.129	R	11.32	0.079	47.0%	0.230	0.314
PCR-RFLP	21	5029/6051	1.06	0.87-1.30	0.534	R	39.18	0.006	49.0%	0.156	0.290
Taqman	4	1152/2920	0.95	0.73-1.24	0.709	F	2.58	0.460	0.0%	0.734	0.974
Sequence	2	285/408	0.94	0.50-1.77	0.843	F	0.96	0.328	0.0%	1.000	
*Arg/Arg vs. Arg/Gln +Gln/Gln*											
Total	27	6,466/9,379	0.99	0.90-1.09	0.869	R	46.67	0.008	44.3%	0.677	0.721
Caucasian	14	2,639/4,768	0.94	0.85-1.04	0.233	F	14.54	0.337	10.6%	0.381	0.380
Asian	9	2,234/2,931	1.04	0.85-1.28	0.687	R	23.77	0.003	66.3%	0.917	0.472
OC	4	663/1,162	0.88	0.55-1.42	0.612	R	15.80	0.001	81.0%	1.000	0.657
Smoking	7	1,039/746	0.73	0.45-1.19	0.206	R	35.56	0.000	83.1%	0.230	0.038
Publication year	7	1898/2765	1.13	0.94-1.36	0.199	R	12.65	0.049	52.6%	0.764	0.225
PCR-RFLP	21	5029/6051	0.99	0.89-1.12	0.928	R	40.73	0.004	50.9%	0.833	0.795
Taqman	4	1152/2920	0.94	0.81-1.09	0.412	F	2.95	0.400	0.0%	0.734	0.734
Sequence	2	285/408	1.17	0.86-1.59	0.308	F	1.37	0.242	27.1%	1.000	

Abbreviations: CI, confidence interval; OR, odds ratio; OC, oral cancer; R: random-effects model; F: fixed-effects model.

#### XRCC1 Arg194Trp polymorphism on HNC risk in total population

**Figure 2:**
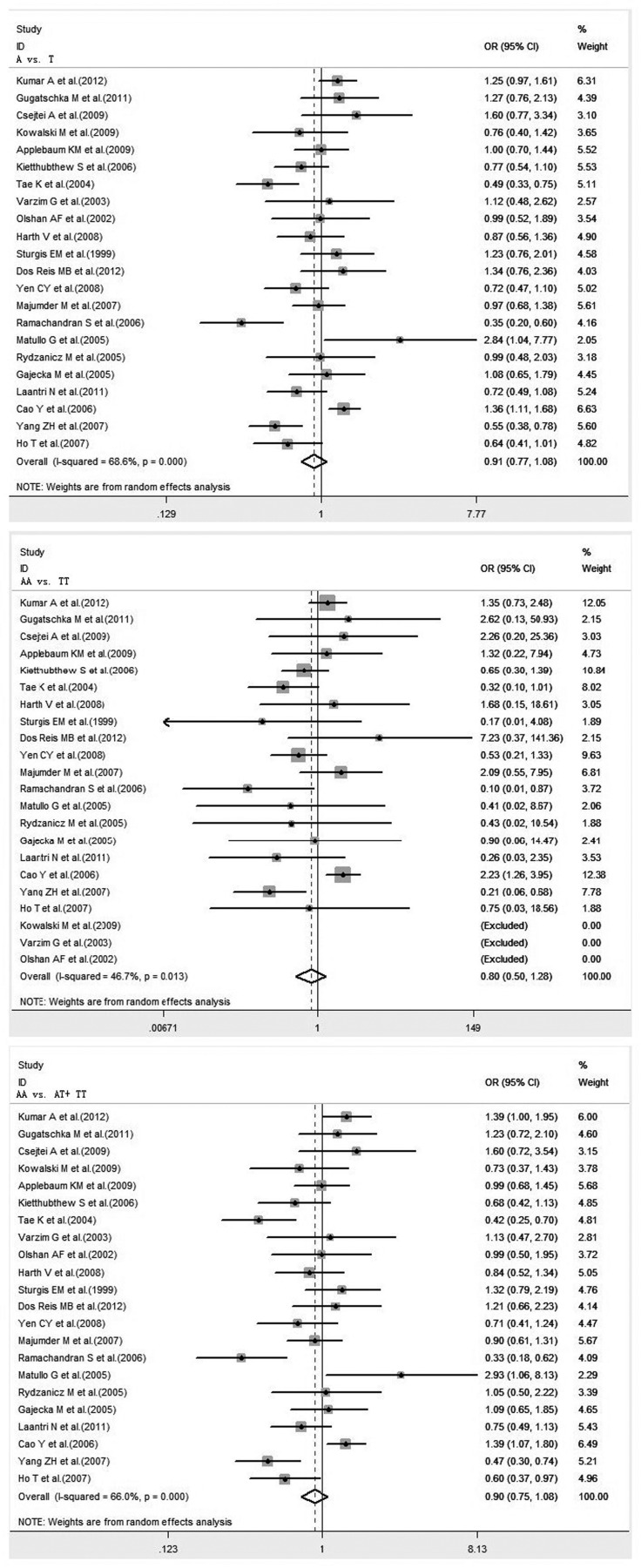
Forest plot of HNC risk associated with XRCC1 Arg194Trp gene polymorphism under all genetic models in total population.

A total of 22 studies, including 4,487 cases and 6,873 controls, examining the association between XRCC1 Arg194Trp polymorphism and HNC risk were reviewed. There was significant difference in the frequency of the XRCC1 Arg194Trp polymorphism between Caucasians and Asians (34.42% vs. 12.27%, P<0.001). The pooled ORs for total population showed no evidence of a significant association between the variant genotypes of XRCC1 Arg194Trp and the risk of HNC in any genetic model. Significant heterogeneity was found in all genetic models. The forest plot is shown in Figure 2.

#### XRCC1 Arg194Trp polymorphism on HNC risk in a specific population

Stratified analysis by ethnicity was performed in order to determine the source of heterogeneity among the studies. No significant association of HNC risk with XRCC1 Arg194Trp polymorphism was detected in Asians and Caucasians under any genetic model (Figures S1-S2). Significant differences between-study heterogeneities were found in the Asians, but they were not found in the Caucasians.

Oral cancer (OC) is the most common form of HNC and it is responsible for more than 90% of head and neck cancers [56]. Consequently, we performed a stratified analysis to investigate the relationship between XRCC1 Arg194Trp polymorphism and OC susceptibility. Six studies, including 915 cases and 1,412 controls, evaluating the association between OC risk and XRCC1 variant genotypes were included (Table 1). No significant association between the XRCC1 Arg194Trp polymorphism and risk of OC was found in the allelic genetic model and the homozygote comparison, but a significant association was found for the recessive model (Figure S3). Between-study heterogeneities were detected in the allelic genetic model and the recessive model, but it was not found to be significant in the homozygote comparison.

**Figure 3:**
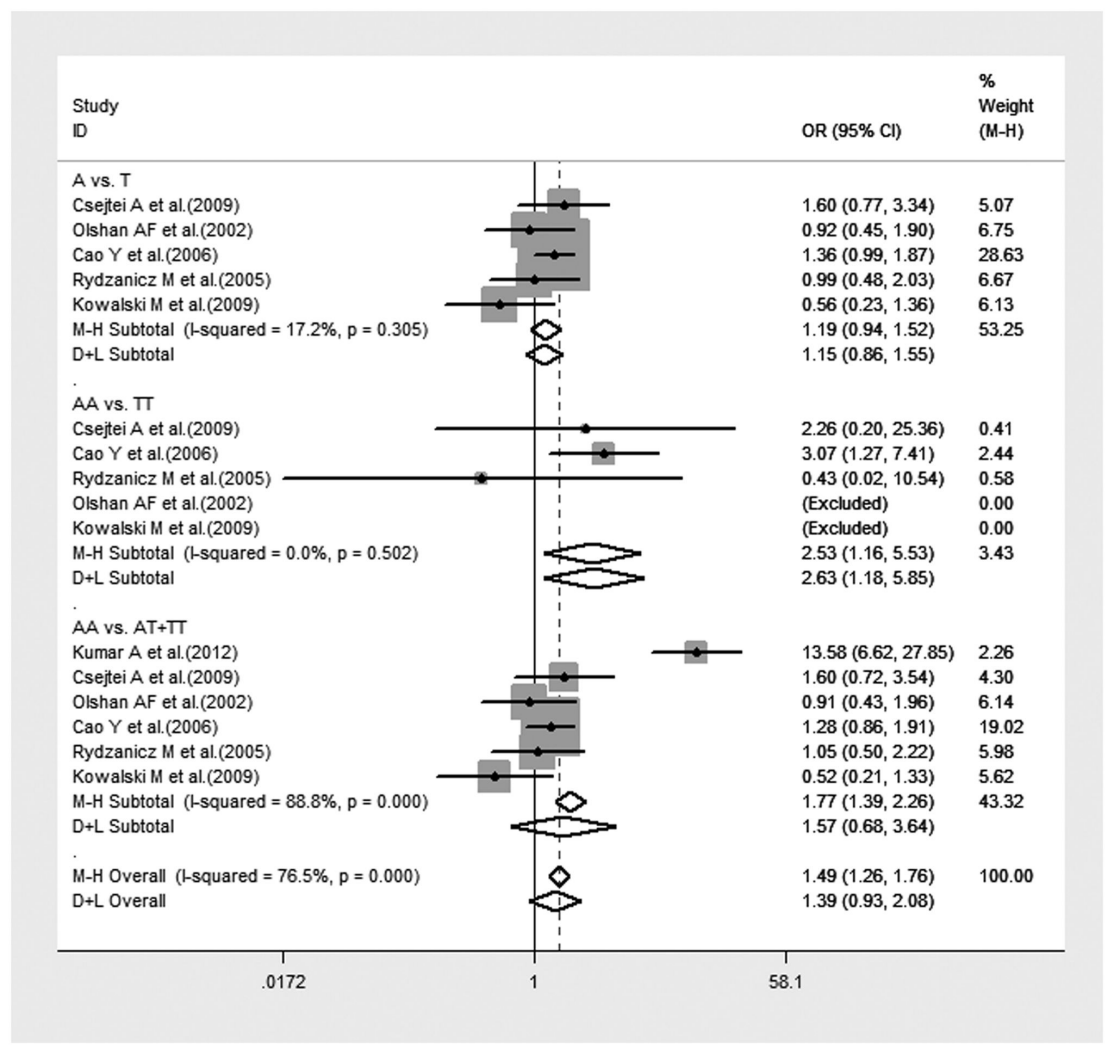
Forest plot of HNC risk associated with interaction between XRCC1 Arg194Trp polymorphism and smoking under all genetic models.

Many studies have demonstrated that the interaction between XRCC1 polymorphism and environmental toxins could influence the risk of HNC. Considering that smoking is a major aspect of environmental toxins, we performed a subgroup analysis of six studies to investigate the influence that the interaction of tobacco smoke with XRCC1 polymorphism has on HNC risk. There was a significant association between the joint effect of smoking with XRCC1 Arg194Trp polymorphism and the risk of HNC under homozygote comparison (Figure 3). No significant association was observed in the allelic genetic model and the recessive model (Figure 3). Heterogeneity among the studies was not remarkable in any genetic model, except for the recessive model.

Flores-Obando et al. conducted a similar meta-analysis that included studies published before 2010. Considering the inconsistent results between the two studies, we decided to perform a stratified analysis by including studies published after 2010. The result showed no significant association was detected between Arg194Trp polymorphism and HNC risk in any genetic model (Figure S4). Between-study heterogeneity was not remarkable in this stratified analysis.

The different genotyping methods used in the literature could cause the different genotyping results. Therefore, we performed a subgroup analysis by genotyping methods to investigate the relationship between Arg194Trp and HNC susceptibility. Neither the PCR-RFLP subgroup nor the TaqMan subgroup detected any significant association in the analyses for all genetic models (Figures S5-S6). Moreover, heterogeneity among the studies was observed in the two stratified analyses under all genetic models, except for the homozygote comparison in the TaqMan subgroup.

#### XRCC1 Arg280His polymorphism on HNC risk in total population

**Figure 4:**
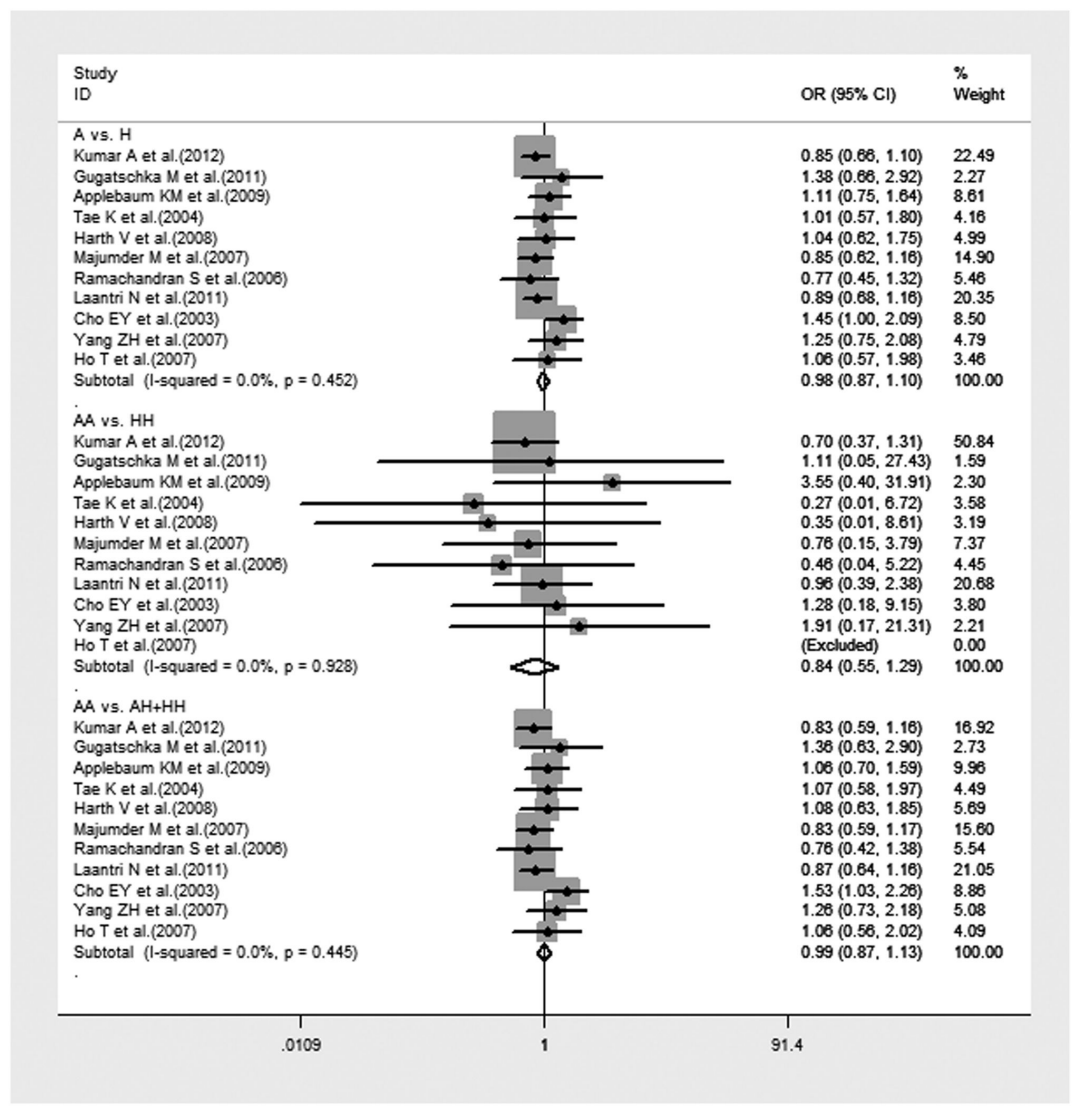
Forest plot of HNC risk associated with XRCC1 Arg280His gene polymorphism under all genetic models in total population.

Eleven studies, including 2,972 cases and 3,714 controls, examining the relationship between XRCC1 Arg280His polymorphism and HNC risk were reviewed. There was significant difference in the frequency of the XRCC1 Arg280His polymorphism between Caucasians and Asians (25.75% vs. 9.04%, P<0.001). There was no significant association of HNC risk with variant genotypes of XRCC1 Arg280His in any genetic model. Significant between-study heterogeneity was absence in all genetic models. The forest plot is shown in Figure 4.

#### XRCC1 Arg280His polymorphism on HNC risk in specific a population

We conducted subgroup analyses by ethnicity, tumor site, publication year, and genotyping method to estimate the relationship between XRCC1 Arg280His variant genotypes and the risk of NHC. However, no significant association was observed in any subgroup under different genetic models, and there was no significant heterogeneity among the studies in any stratified analysis (Figures S7-S12). Only one study evaluated the influence of the interaction between smoking and Arg280His polymorphism on HNC risk; however, we are unable to conduct a further stratified analysis of that study.

#### XRCC1 Arg399Gln polymorphism on HNC risk in total population

**Figure 5:**
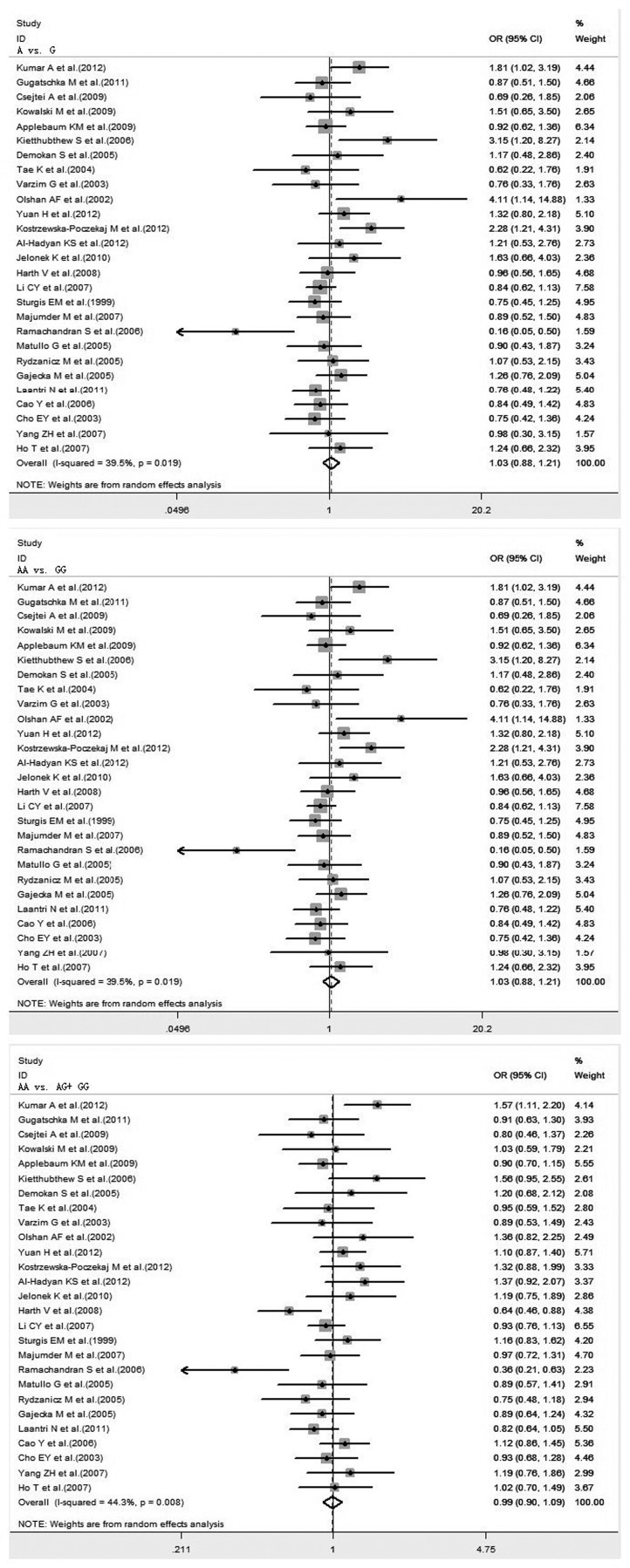
Forest plot of HNC risk associated with XRCC1 Arg399Gln gene polymorphism under all genetic models in total population.

There were 27 studies, including 6,466 cases and 9,379 controls, that examined the association between HNC susceptibility and XRCC1 Arg399Gln polymorphism. There was significant difference in the frequency of the XRCC1 Arg399Gln polymorphism between Caucasians and Asians (41.28% vs. 44.65%, P=0.004). Overall, the association between variant genotypes of XRCC1 Arg399Gln polymorphism and HNC susceptibility was not significant under the allelic genetic model, homozygote comparison, and the recessive model. Between-study heterogeneity was detected in all genetic models. The forest plot is shown in Figure 5.

#### XRCC1 Arg399Gln polymorphism on HNC risk in a specific population

In the subgroup analysis by ethnicity, no significant association between XRCC1 Arg399Gln polymorphism and HNC risk was found in Asians (Figure S13) and Caucasians (Figure S14). Heterogeneity among the studies was not remarkable in Caucasians; however, significant heterogeneity was detected in Asians under all genetic models.

Four studies, including 663 cases and 1,162 controls, were performed on OC population, and there was no significant association between XRCC1 Arg399Gln polymorphism and HNC susceptibility (Figure S15). Between-study heterogeneity was found in all genetic models.

In the stratified analysis by smoking in the allelic genetic model and homozygote comparison, four studies were included and no significant association was found (Figure S16). Seven studies were combined in the recessive model. However, we failed to derive a significant association between HNC risk and Arg399Gln genotype (Figure S16). Heterogeneity among the studies was observed in all genetic models.

In stratified analysis by publication year of literature published from 2010–2012, significant heterogeneity was detected in all genetic models. Moreover, we found no association between Arg399Gln and HNC risk under any genetic model (Figure S17).

In subgroup analysis of genotyping method, PCR-RFLP, TaqMan, and sequence analysis were used in the literature for genotyping of XRCC1 Arg399Gln polymorphism. The results showed no significant association between Arg399Gln and HNC risk in any stratified analysis under different genetic models (Figures S18-S20).

### Publication bias

**Figure 6:**
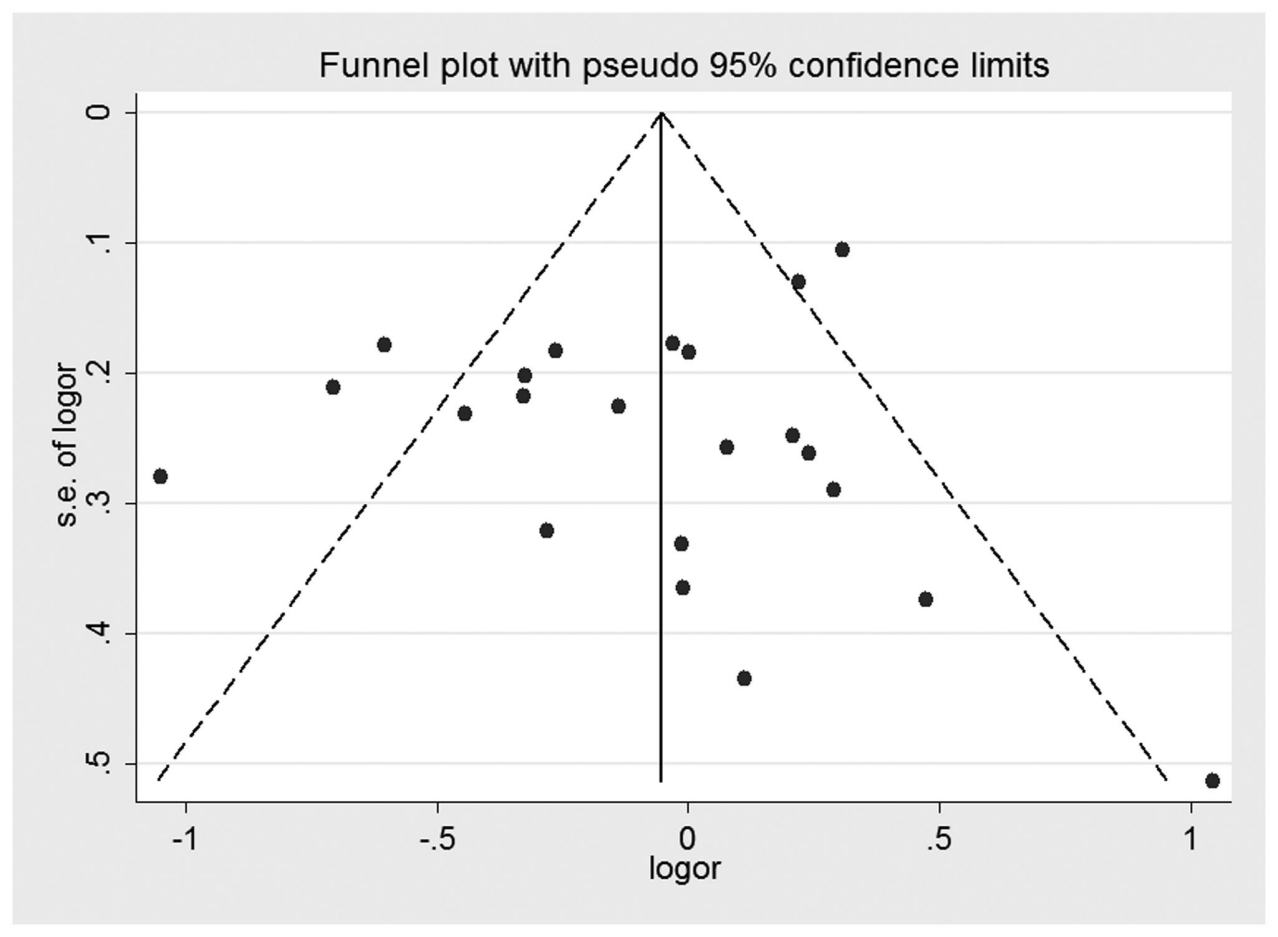
Funnel plot for studies of the association of HNC risk and XRCC1 Arg194Trp gene polymorphism under an allelic genetic model.

**Figure 7:**
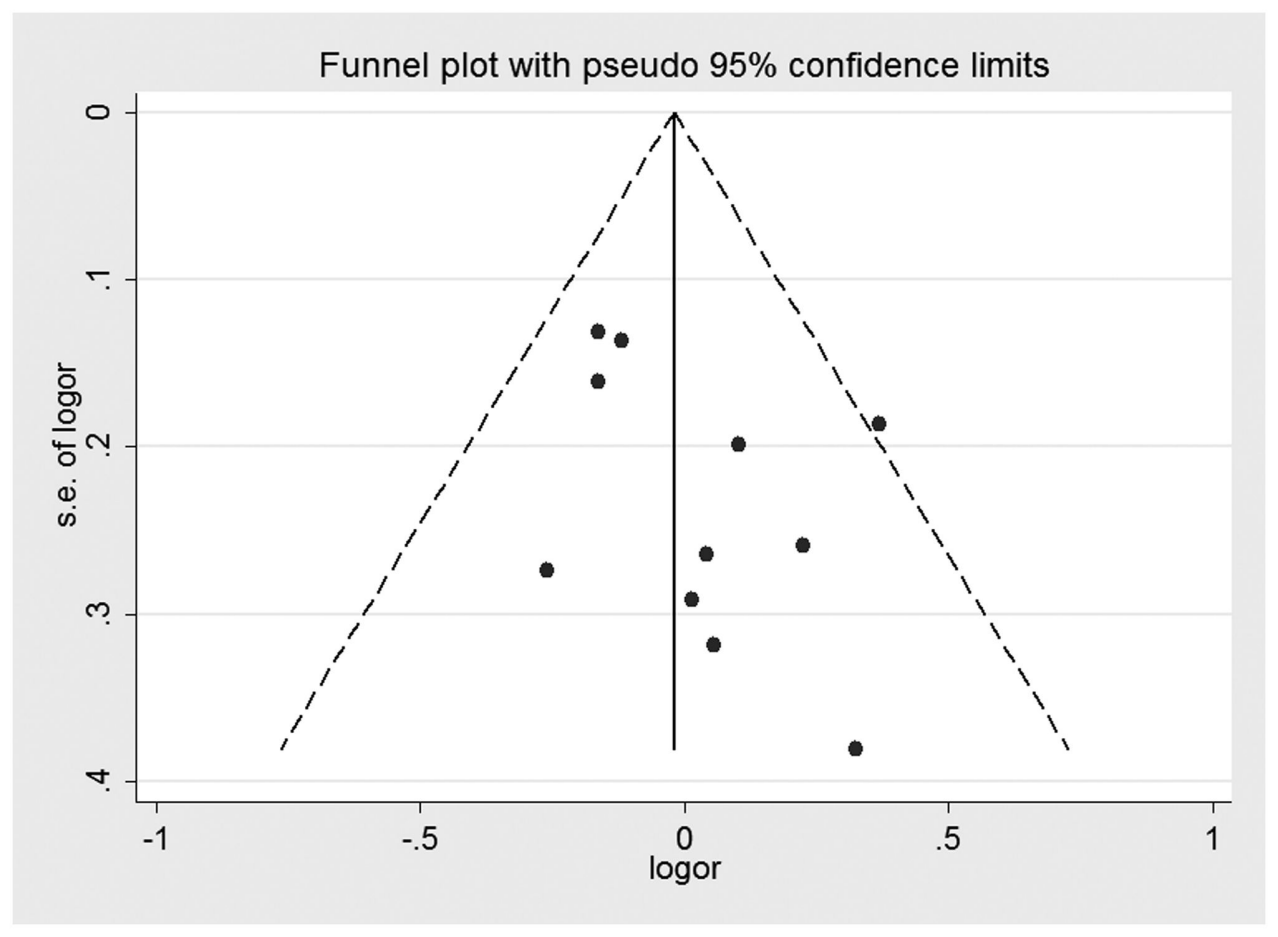
Funnel plot for studies of the association of HNC risk and XRCC1 Arg280His gene polymorphism under an allelic genetic model.

**Figure 8:**
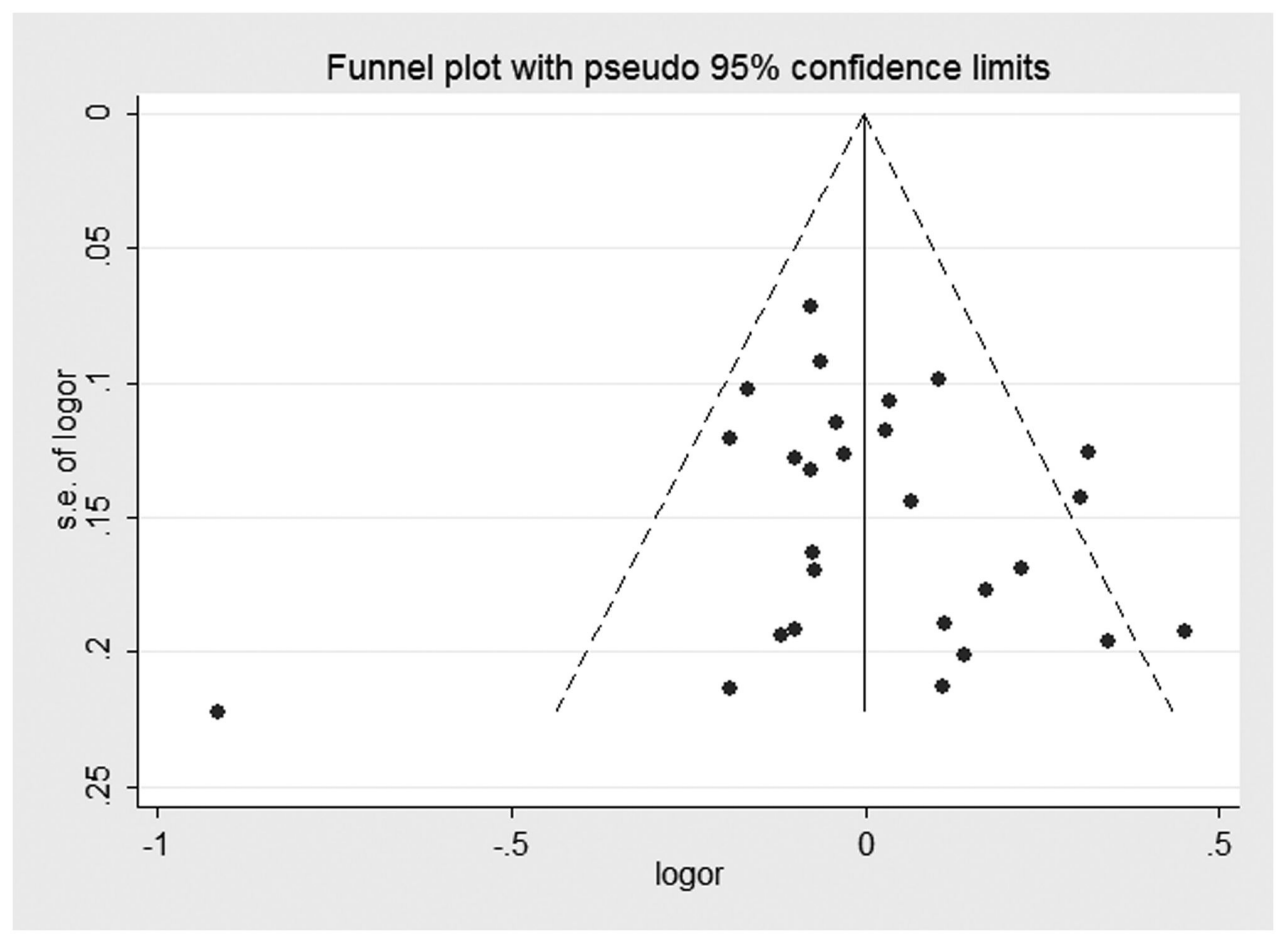
Funnel plot for studies of the association of HNC risk and XRCC1 Arg399Gln gene polymorphism under an allelic genetic model.

Both Begg’s test and Egger’s test were performed to assess the publication bias of the literature. Visual analysis of the funnel plots did not present any evidence of obvious asymmetry for any genetic model in the overall meta-analyses of XRCC1 Arg194Trp, Arg280His, and Arg399Gln (Figures 6-8). However, obvious evidence of publication bias was revealed in the XRCC1 Arg194Trp Asian group under all genetic models. In XRCC1 Arg399Gln smoking stratified analysis, potential publication bias was not revealed in Begg’s test under any genetic model, but it was presented in the Egger’s test. Neither the Begg’s test nor the Egger’s test detected any obvious evidence of publication bias in other stratified analyses for all genetic models (Table 3).

## Discussion

DNA repair mechanisms play a critical role in the protection of cells from DNA damage and in the maintenance of genomic integrity. The protein encoded by the XRCC1 gene is a scaffolding protein that associates with DNA ligase I, DNA ligase III, polynucleotide kinase (PNK), DNA polymerase β, and poly polymerase, which are parts of the DNA repair system. The interaction of XRCC1 with DNA ligase III could increase the endocellular stability of ligase. The joint effects of XRCC1 and PNK stimulate the 5’-kinase and 3’-phospatase activities. All of these conditions promote the repair of DNA. Therefore, sequence variation in the XRCC1 gene is suggested to alter cancer’s susceptibility. The most common variant genotypes of XRCC1, including the Arg194Trp, Arg399Gln, and Arg280His genes, are described and a number of studies have investigated the genetic effect of the XRCC1 Arg194Trp, Arg280His, and Arg399Gln polymorphisms on HNC susceptibility with inconsistent results. This diversity motivates the current updated meta-analysis that may help us to explore a more robust estimate of the effect of XRCC1 polymorphism on the risk of HNC. In the present meta-analysis of 6,719 cases and 9,627 controls, no evidence of a significant association between HNC susceptibility and any type of XRCC1 variant genotype was detected.

**Figure 9:**
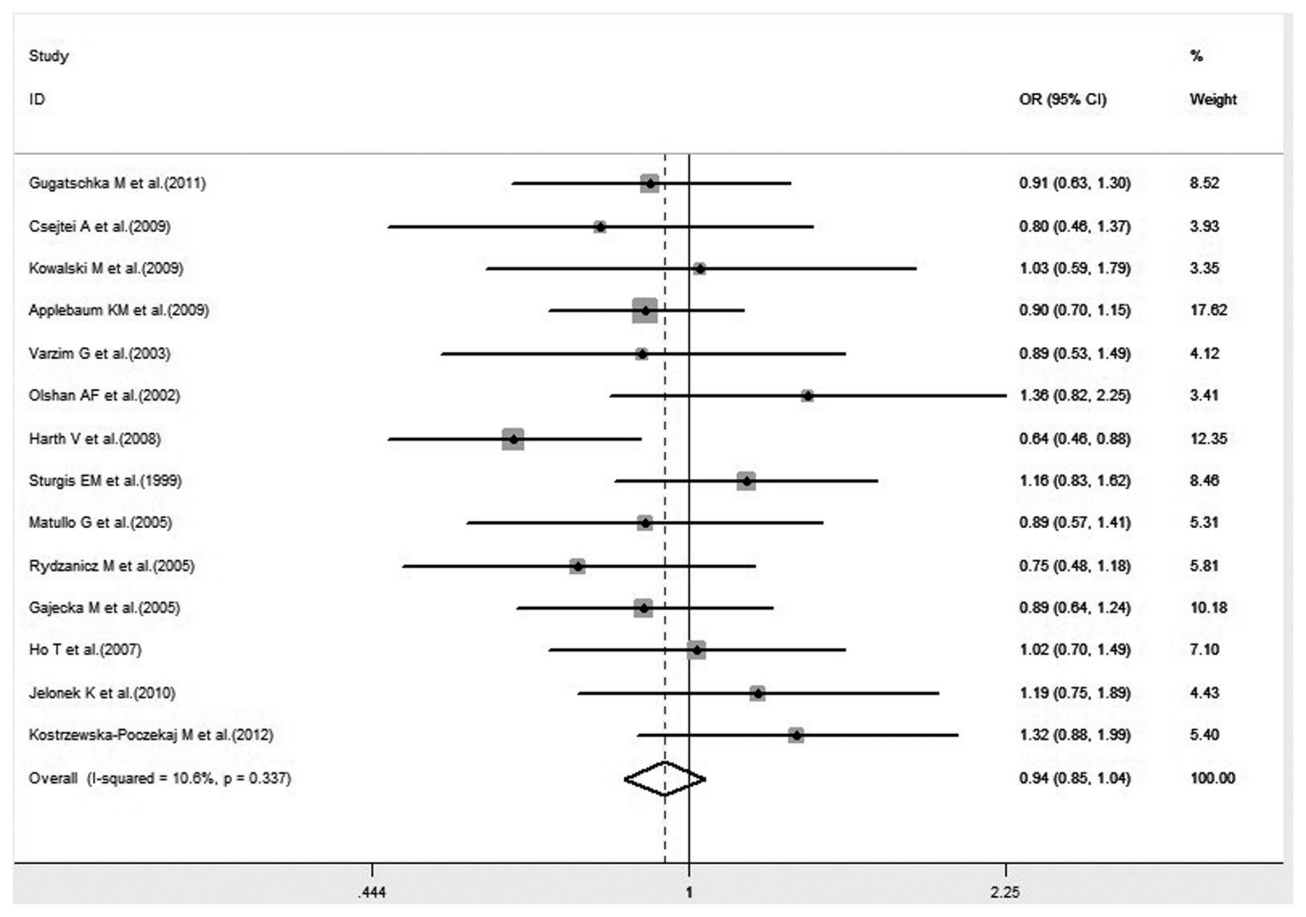
Forest plot of HNC risk associated with XRCC1 Arg399Gln gene polymorphism under recessive genetic models on Caucasians.

**Figure 10:**
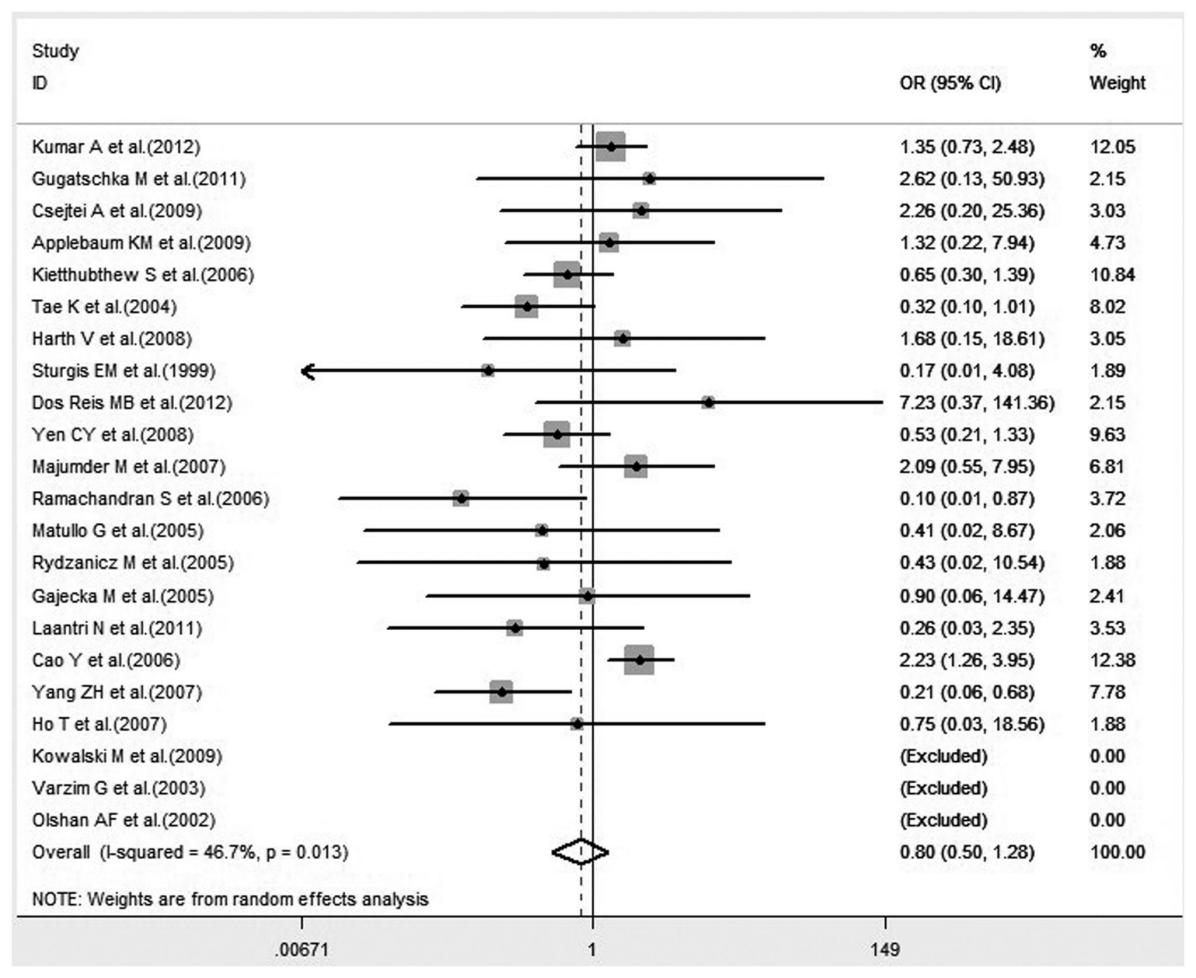
Forest plot of HNC risk associated with XRCC1 Arg194Trp gene polymorphism under a homozygote comparison in total population.

**Figure 11:**
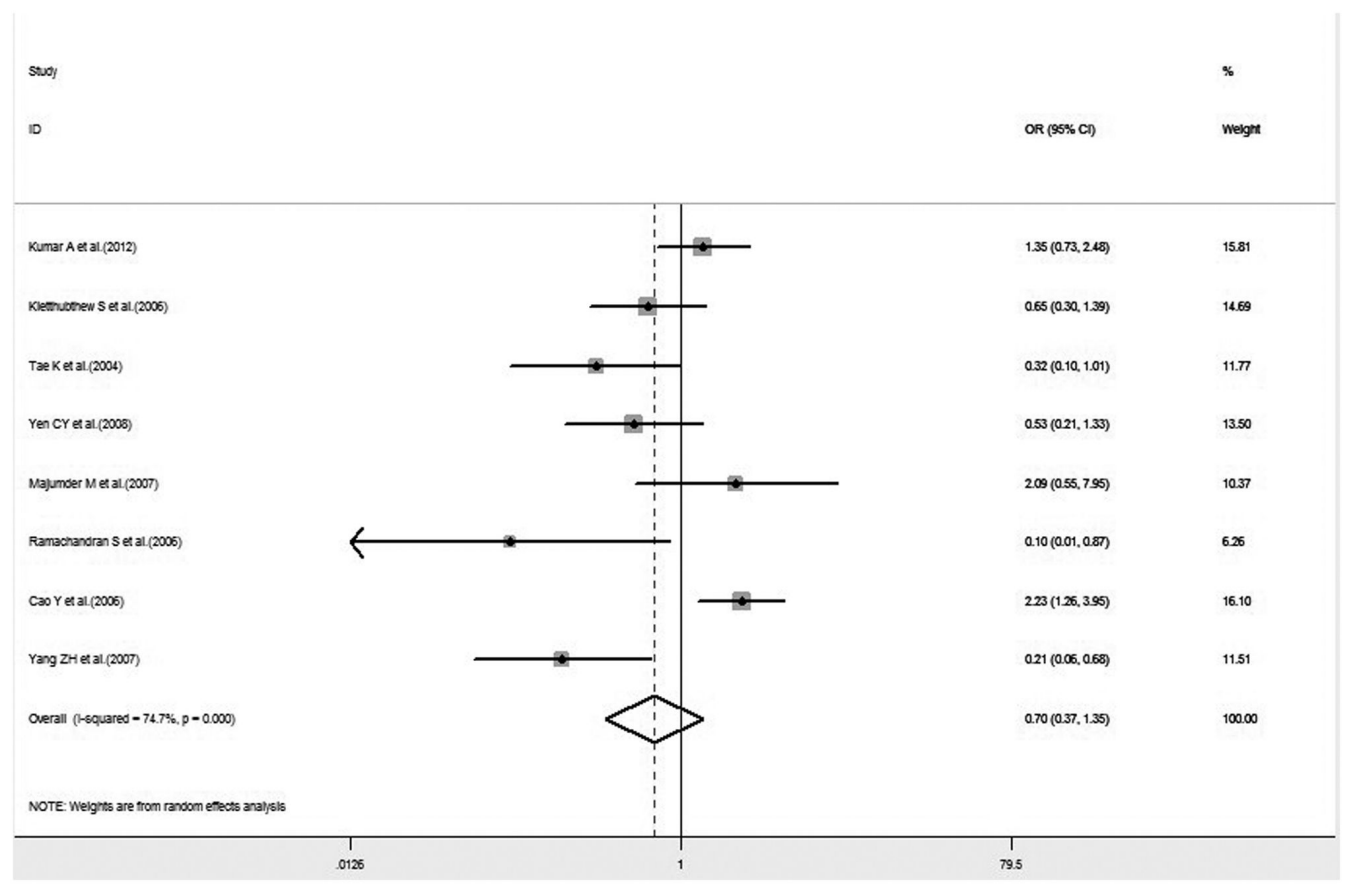
Forest plot of HNC risk associated with XRCC1 Arg194Trp gene polymorphism under a homozygote comparison on Asians.

A previous meta-analysis, conducted in 2010 by Flores-Obando et al., evaluated the relationship between XRCC1 polymorphisms and the risk of HNC based on 15 publications including 2,330 cases and 3,834 controls for Arg194Trp, four publications including 879 cases and 926 controls for Arg280His, and 15 studies including 3,582 cases and 5,347 controls for Arg399Gln polymorphism. We updated this meta-analysis by adding the sample sizes. A total of 22 studies, including 4,487 cases and 6,873 controls, evaluated the association between the XRCC1 Arg194Trp gene and HNC risk; 11 studies, including 2,972 cases and 3,714 controls, evaluated the association between Arg280His and HNC risk; and 27 studies, including 6,466 cases and 9,379 controls, evaluated the association between Arg399Gln polymorphism and HNC risk. There are some discrepancies between the Flores-Obando et al. meta-analysis and ours. A marginal association between XRCC1 Arg399Gln polymorphism and HNC risk was detected under the recessive genetic model on Caucasians in the meta-analysis conducted by Flores-Obando et al., but it was not found in ours. These diverse results may, generally, be due to the differences in the studies included in the meta-analysis. Nasopharyngeal carcinoma (NPC) is one type of HNC, which has a striking geographic and ethnic distribution, with particularly high rates observed among Asians. The literature on NPC was not included in the Flores-Obando et al. study, but it was included in ours. In the above-mentioned stratified analysis result, seven articles were shared the Flores-Obando et al. study and our study, and our study included an additional seven articles, including newly published literature and NPC literature. The results of these seven studies account for 54.94% weight (Figure 9), which caused the discrepancy between these two meta-analyses. In the assessment of the effect of XRCC1 Arg194Trp variant genotypes on HNC susceptibility, the meta- OR conducted by Flores-Obando et al. found a significant association between the Arg194Trp variant and HNC risk for homozygote comparison in the overall population and in the Asian group, which was not detected in our meta-analysis. Similarly, in our study, an additional nine studies and four studies, comprised of recently published research and NPC studies, included in the overall population group and the Asian group, respectively. The results of these additional studies account for 53.46% and 53.79% weight (Figures 10-11), respectively. In the controls in a study conducted by Demokan et al., genotype distribution in Arg194Trp deviated from HWE, which was excluded in our analysis of Arg194Trp polymorphism; however, it was included in the Flores-Obando et al. article. Furthermore, both our study and the Flores-Obando et al. study included two studies by Majumder et al. Majumder et al.’s most recent publication was included in our study, but Majumder et al.’s research published in 2005 was included in the Flores-Obando et al. meta-analysis. These factors lead to the different conclusion. Many other relationships were not described in the Flores-Obando et al. article. Moreover, we carried out some independent and original subgroup analyses. Subgroup analysis of smoking was not performed in the Flores-Obando’s study, but it was included in this meta-analysis. We also conducted stratified analyses by genotyping methods and publication year, and all the results revealed no association between XRCC1 polymorphism and cancer risk. Therefore, our meta-analysis has stronger evidence to clarify the associations.

The relationship between HNC susceptibility and variant genotypes of XRCC1 might be affected by the tumor sites. Accordingly, we also performed stratified analysis in the oral cancer group. The result of this subgroup analysis showed no evidence for a significant association between XRCC1 polymorphism and the risk of OC in any genetic model, except for the Arg194Trp variant in the recessive model. These results generally agreed with the meta-analysis conducted by Zhou et al. [57], but there were still several differences. Two studies by Sturgis et al. [45] and Matullo et al. [49], which included oral cavity, pharynx, and larynx cancer cases, were not excluded from the Zhou et al. study. The two studies [31,48] were conducted by the same first author and the patient population was obtained from the same hospital; as a result, there is a suspicion that the findings are a duplication of a previous publication. Therefore, only one study met the inclusion criteria to participate in our research.

**Figure 12:**
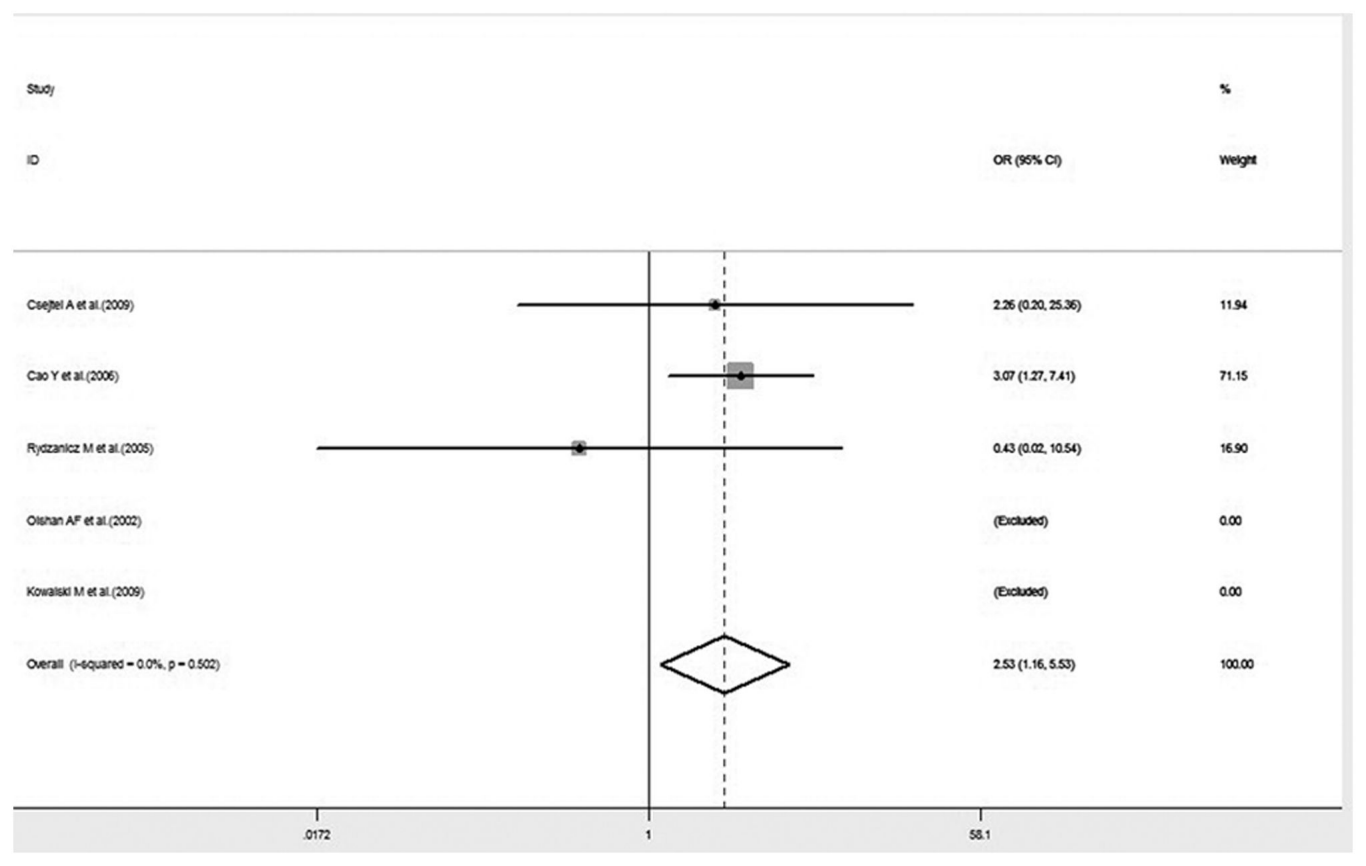
Forest plot of HNC risk associated with interaction between XRCC1 Arg194Trp polymorphism and smoking under a homozygote comparison.

Recent studies have reported on the associated risk of XRCC1 polymorphism cross lifestyle factors in the progression of head and neck cancer. Cigarette smoking is a major subject of investigation in various cancers. In 1997, the World Health Organization reported that there were 1.1 billion smokers worldwide and smoking-related cancers accounted for 22% of all cancers. Hence, subgroup analysis to estimate the interaction between the genotypes of XRCC1 Arg194Trp and Arg399Gln and smoking on HNC risk was performed. The results showed that the interaction of smoking and Arg399Gln variant genotypes displayed no statistical significance in all three genetic models. A significant association between the joint effect of smoking and XRCC1 Arg194Trp polymorphism and HNC susceptibility was detected under a homozygote comparison (OR= 2.53, 95% CI= 1.16-5.53, P= 0.020), but no statistical significance was revealed in the other genetic models. In the forest plot of HNC risk associated with the interaction between smoking and Arg194Trp, the result of a study by Cao et al. accounted for 71.15% weight (Figure 12), which may mean that XRCC1 Arg194Trp variants are nominally associated with HNC susceptibility in smokers even at a lenient threshold for statistical significance (P= 0.05). Hence, careful consideration is needed for the lack of signals with strong credibility that emerged from this subgroup analysis. The results suggest that XRCC1 Arg194Trp polymorphism may have a small involvement in the pathogenesis of HNC in smokers.

Between-study heterogeneity is a well-known problem that is unavoidable. In our meta-analysis, heterogeneity was detected in the Arg194Trp and Arg399Gln polymorphism total population groups (Table 3). The source of heterogeneity may arise from many aspects, such as the region of study, the sample size of the case and the control group, and the genotyping method. In order to explain the main reasons for the heterogeneity across studies, stratified analyses by ethnicity and genotyping method were performed. The results showed that in both the Arg194Trp group and the Arg399Gln group, significant heterogeneity was observed in the Asian population subgroup and in the PCR-RFLP analysis subgroup under all different genetic models. This signifies that the source of total population group heterogeneity may come from different races and different genotyping methods.

Publication bias is a well-known problem that was not found by funnel plot for the overall meta-analyses of the XRCC1 polymorphisms (Figures 6-8). However, we found a potential publication bias in the Arg194Trp Asians stratified analysis and the Arg399Gln smoking stratified analysis. The reasons for this could arise from many aspects. For instance, our meta-analysis took into consideration only fully published studies. Positive results tend to be accepted by journals. In addition, language bias may also have existed.

Some limitations should be considered in our meta-analysis. First, some of the included studies in our meta-analysis contained a smaller sample size, which might result in a lack of ability to detect the possible risk for XRCC1 polymorphism. Second, due to the limited number of studies, subgroup analysis was not performed in Africans [12]. Third, this study was based on unadjusted estimates, while a more precise analysis could be performed if individual data were available.

Despite of the limitations mentioned above, the results of the current meta-analysis suggest that XRCC1 Arg194Trp, Arg280His, and Arg399Gln polymorphism is not involved in HNC susceptibility. In addition, further studies evaluating the effect of gene-gene and gene-environment interactions on these gene polymorphisms with HNC susceptibility are required, especially in an African population.

## Supporting Information

Figure S1 Forest plot of HNC risk associated with XRCC1 Arg194Trp gene polymorphism under all genetic models on Asians.
(DOC)

Figure S2 Forest plot of HNC risk associated with XRCC1 Arg194Trp gene polymorphism under all genetic models on Caucasians.
(DOC)

Figure S3 Forest plot of HNC risk associated with XRCC1 Arg194Trp gene polymorphism under all genetic models in oral cancer population.
(DOC)

Figure S4 Forest plot of HNC risk associated with XRCC1 Arg194Trp gene polymorphism under all genetic models in the stratified analysis by studies published after 2010.
(DOC)

Figure S5 Forest plot of HNC risk associated with XRCC1 Arg194Trp gene polymorphism under all genetic models in using PCR-RFLP analysis.
(DOC)

Figure S6 Forest plot of HNC risk associated with XRCC1 Arg194Trp gene polymorphism under all genetic models in using TaqMan analysis.
(DOC)

Figure S7 Forest plot of HNC risk associated with XRCC1 Arg280His gene polymorphism under all genetic models on Asians.
(DOC)

Figure S8 Forest plot of HNC risk associated with XRCC1 Arg280His gene polymorphism under all genetic models on Caucasians.
(DOC)

Figure S9 Forest plot of HNC risk associated with XRCC1 Arg280His gene polymorphism under all genetic models in oral cancer population.
(DOC)

Figure S10 Forest plot of HNC risk associated with XRCC1 Arg280His gene polymorphism under all genetic models in the stratified analysis by studies published after 2010.
(DOC)

Figure S11 Forest plot of HNC risk associated with XRCC1 Arg280His gene polymorphism under all genetic models in using PCR-RFLP analysis.
(DOC)

Figure S12 Forest plot of HNC risk associated with XRCC1 Arg280His gene polymorphism under all genetic models in using TaqMan analysis.
(DOC)

Figure S13 Forest plot of HNC risk associated with XRCC1 Arg399Gln gene polymorphism under all genetic models on Asians.
(DOC)

Figure S14 Forest plot of HNC risk associated with XRCC1 Arg399Gln gene polymorphism under all genetic models on Caucasians.
(DOC)

Figure S15 Forest plot of HNC risk associated with XRCC1 Arg399Gln gene polymorphism under all genetic models in oral cancer population.
(DOC)

Figure S16 Forest plot of HNC risk associated with interaction between XRCC1 Arg399Gln polymorphism and smoking under all genetic models.
(DOC)

Figure S17 Forest plot of HNC risk associated with XRCC1 Arg399Gln gene polymorphism under all genetic models in the stratified analysis by studies published after 2010.
(DOC)

Figure S18 Forest plot of HNC risk associated with XRCC1 Arg399Gln gene polymorphism under all genetic models in using PCR-RFLP analysis.
(DOC)

Figure S19 Forest plot of HNC risk associated with XRCC1 Arg399Gln gene polymorphism under all genetic models in using TaqMan analysis.
(DOC)

Figure S20 Forest plot of HNC risk associated with XRCC1 Arg399Gln gene polymorphism under all genetic models in using sequence analysis.
(DOC)

Supplement S1 PRISMA Flowchart.
(DOC)

Supplement S2 PRISMA Checklist.
(DOC)
