# Supplementary material for: DNA Repair Gene XRCC1 Polymorphisms and Head and Neck Cancer Risk: An Updated Meta-Analysis Including 16344 Subjects
Source: PLoS One. 2013 Sep 23;8(9):e74059. doi: 10.1371/journal.pone.0074059 (PMC3781168; doi:10.1371/journal.pone.0074059)

Figure S1: Forest plot of HNC risk associated with XRCC1 Arg194Trp gene polymorphism under all genetic models in Asians.


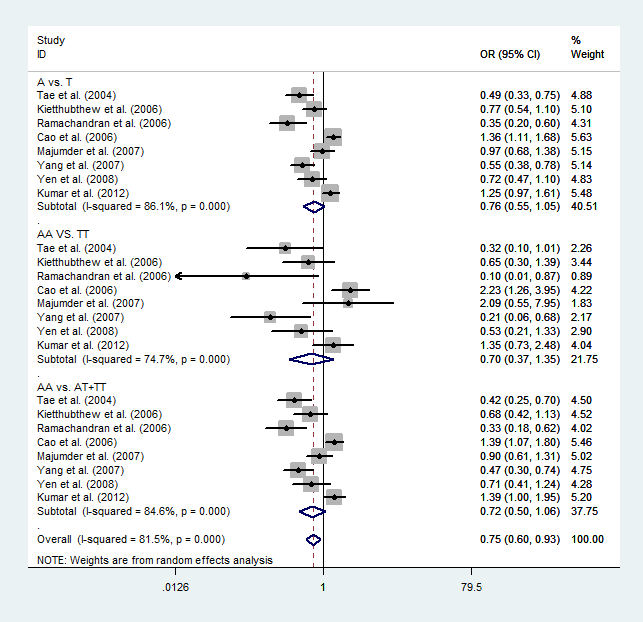

Supplement: Figure S1 — Forest plot of HNC risk associated with XRCC1 Arg194Trp gene polymorphism under all genetic models on Asians. (DOC) [file pone.0074059.s001.doc]
