# Supplementary material for: DNA Repair Gene XRCC1 Polymorphisms and Head and Neck Cancer Risk: An Updated Meta-Analysis Including 16344 Subjects
Source: PLoS One. 2013 Sep 23;8(9):e74059. doi: 10.1371/journal.pone.0074059 (PMC3781168; doi:10.1371/journal.pone.0074059)

Figure S15: Forest plot of HNC risk associated with XRCC1 Arg399Gln gene polymorphism under all genetic models in oral cancer population.


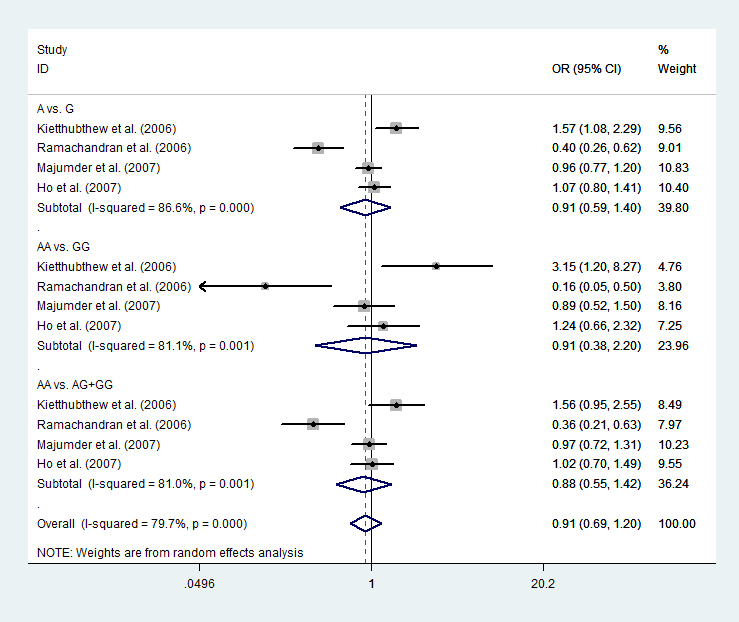

Supplement: Figure S15 — Forest plot of HNC risk associated with XRCC1 Arg399Gln gene polymorphism under all genetic models in oral cancer population. (DOC) [file pone.0074059.s015.doc]
