# Supplementary material for: DNA Repair Gene XRCC1 Polymorphisms and Head and Neck Cancer Risk: An Updated Meta-Analysis Including 16344 Subjects
Source: PLoS One. 2013 Sep 23;8(9):e74059. doi: 10.1371/journal.pone.0074059 (PMC3781168; doi:10.1371/journal.pone.0074059)
Supplement: Supplement S1 — PRISMA Flowchart. (DOC) [file pone.0074059.s021.doc]

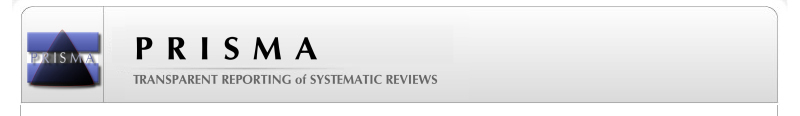
**PRISMA 2009 Flow Diagram**

**Screening**

**Included**

**Eligibility**

**Identification**

Records identified through database searching
(n = 38)

Additional records identified through other sources
(n = 0)

Records after duplicates removed
(n = 36)

Records screened
(n = 36)

Records excluded
(n = 2)

Full-text articles assessed for eligibility
(n = 34)

Full-text articles excluded, with reasons
(n = 5)

Studies included in qualitative synthesis
(n = 29)

Studies included in quantitative synthesis (meta-analysis)
(n = 29)
